# P-1723. Sensitivity of Antigen Testing for Histoplasmosis and Blastomycosis in Bronchoalveolar Lavage (BAL) and Cerebrospinal Fluid (CSF) Versus Urine and Serum

**DOI:** 10.1093/ofid/ofaf695.1894

**Published:** 2026-01-11

**Authors:** Dane Granger, Elitza Theel

**Affiliations:** Mayo Clinic, Rochester, Minnesota; Mayo Clinic, Rochester, Minnesota

## Abstract

**Background:**

Detecting *Histoplasma* (HP) and *Blastomyces* (BM) antigen (Ag) in urine (U) and serum (S) is an essential diagnostic tool for these pathogens. Labs have added bronchoalveolar lavage (BAL) and cerebrospinal fluid (CSF) as additional HP and BM Ag testing sources. We evaluated the sensitivity of Ag detection in U and S in patients with detected HP or BM Ag in BAL or CSF by enzyme immunoassay (EIA).

**Methods:**

14 patients with HP or BM Ag-positive results made up the CSF cohort. The Ag concentrations were below the limit of quantification (< LoQ, *N*=3), above the limit of quantification ( > LoQ, *N*=1), and 0.3 to 9.5 ng/mL (x̄ = 2.3 ng/mL, *N*=10).

The BM Ag-positive in BAL group included 24 patients, with concentrations of < LoQ (*N*=4), >LoQ (*N*=5), and 0.3 to 11.3 ng/mL (x̄ = 3.5 ng/mL, *N*=15).

HP Ag-positive in BAL comprised 36 patients, with 3 < LoQ, 10 >LoQ, and 23 ranging from 0.8 to 16.9 ng/mL (x̄ = 4.7 ng/mL).

16 patients in the BAL cohorts were Ag-positive for HP and BM, demonstrating the inability to discriminate between these dimorphic fungi through Ag testing.

All patients were checked for routine clinical HP and BM EIA Ag test results on U and S. HP BAL patients were also investigated for cross-reacting galactomannan (GM; *Aspergillus* EIA in S or BAL) and (1→3)-β-d-glucan (BDG; Fungitell EIA in S) antigen test orders. All additional test results over 14 days before or after CSF or BAL Ag testing were excluded.

**Results:**

100% (14/14), 96% (22/23), and 83% (30/36) of HP/BM CSF, BM BAL, and HP BAL Ag-positive patients were also positive in U and/or S, when both additional sources were ordered, respectively. Of the 6 HP BAL patients without positive U or S results, 3 had GM and BDG testing performed. All 3 had high signal GM and/or BDG results in S and/or BAL. In conjunction with low HP Ag levels in BAL (1.0 to 1.53), these findings suggest cross-reactivity in the HP BAL Ag test. Classifying these 3 patients as HP BAL false-positive shows a positive agreement of 91% (30/33) between HP BAL and HP U/S.

**Conclusion:**

HP and BM CSF Ag testing showed no increase in sensitivity when U and S were tested. Minimal increases in sensitivity were observed in HP and BM BAL versus U and S. However, false-positive results in BAL due to cross-reactivity with GM and BDG cannot be ruled out, suggesting limited utility of the invasive CSF and BAL sources for HP and BM EIA Ag testing.

**Disclosures:**

All Authors: No reported disclosures

